# Elevated ceramides 18:0 and 24:1 with aging are associated with hip fracture risk through increased bone resorption

**DOI:** 10.18632/aging.102389

**Published:** 2019-11-01

**Authors:** Beom-Jun Kim, Jin Young Lee, So Jeong Park, Seung Hum Lee, Su Jung Kim, Hyun Ju Yoo, Sarah I. Rivera De Pena, Meghan McGee-Lawrence, Carlos M. Isales, Jung-Min Koh, Mark W. Hamrick

**Affiliations:** 1 Division of Endocrinology and Metabolism, Asan Medical Center, University of Ulsan College of Medicine, Seoul, South Korea; 2 Asan Institute for Life Sciences, Seoul, South Korea; 3 Biomedical Research Center, Asan Institute for Life Sciences, Asan Medical Center, University of Ulsan College of Medicine, Seoul, South Korea; 4 School of Medicine, Universidad Central Del Caribe, Bayamon, Puerto Rico; 5 Medical College of Georgia, Augusta University, Augusta, GA 30912, USA

**Keywords:** ceramides, osteoclastogenesis, bone resorption, hip fracture, aging

## Abstract

We assessed whether circulating ceramides, which play a role in a number of degenerative changes with aging, significantly differed according to fragility hip fracture (HF) status. We also performed a human study using bone marrow (BM) aspirates, directly reflecting the bone microenvironment, in addition to *in vitro* experiments. Peripheral blood and BM samples were simultaneously collected from 74 patients 65 years or older at hip surgery for either HF (*n* = 28) or for other causes (*n* = 46). Ceramides were measured by liquid chromatography-tandem mass spectrometry. Age was correlated positively with circulating C16:0, C18:0, and C24:1 ceramide levels. Patients with fragility HF had 21.3%, 49.5%, 34.3%, and 22.5% higher plasma C16:0, C18:0, C18:1, and C24:1 ceramide levels, respectively, than those without HF. Higher C16:0, C18:0, C18:1, and C24:1 ceramide levels were positively related to bone resorption markers in both blood and BM samples. Furthermore, *in vitro* studies showed that C18:0 and C24:1 ceramides directly increased osteoclastogenesis, bone resorption, and expression levels of osteoclast differentiation markers. These results suggested that the association of increased ceramides, especially C18:0 and C24:1, with adverse bone phenotypes in elderly people could be explained mainly by the increase in osteoclastogenesis and bone resorption.

## INTRODUCTION

Hip fracture (HF) is a major cause of morbidity and mortality among the elderly. Approximately 40% of those suffering HF will end up in a nursing home, and 20% will never walk again. The one-year mortality rate for HF at age 70 is up to 30%. Thus, HF is a significant public health burden, and its incidence continues to increase in countries worldwide [1]. The causes of HF are complex, but falling is the primary etiological factor in 90% of cases. Postural instability, reduced gait speed, and generalized frailty in older adults are all associated with falls and fractures. Recently, it has been shown that the sphingolipid ceramide (known to induce cell senescence and cell death) [2, 3] is significantly elevated in the serum of older adults with lower cardiorespiratory fitness [4] and gait speed [5], a higher risk of cardiovascular disease [6], and memory impairment [7]. In addition, plasma ceramide is known to increase with age in women, and plasma ceramide levels are negatively correlated with serum estradiol [8]. Therefore, increasing circulating ceramide levels with age are likely to have a role in a number of degenerative changes observed in older adults.

Not surprisingly, ceramides appear to be involved in bone cell survival and cell death. Ceramide accumulation in osteoblasts suppresses bone formation [9], and ceramide can induce cell death in osteoblasts directly [10]. Ceramides are known as second messengers that can stimulate cell death downstream of inflammatory cytokines, such as tumor necrosis factor-α (TNF-α) [11], and osteoblast apoptosis in response to TNF-α is thought to be mediated at least partly by ceramide [12, 13]. Several forms of ceramide exist, including short, medium, long, and very long chain ceramides, which differ from each other primarily in their acyl chain lengths. Very long chain ceramides are linked with mitochondrial damage and cell death [14], and we recently showed that very long chain C24:1 ceramide is elevated in serum extracellular vesicles with age and can induce senescence in human bone marrow (BM) stromal cells (BMSCs) [3]. However, despite the possible effect of increased ceramides with age on bone metabolism, to our knowledge no clinical studies exist relating ceramides to osteoporosis-related phenotypes. Here we assessed whether blood ceramide levels differed significantly according to fragility HF status in older adults and also performed a human study using BM samples, directly reflecting the bone microenvironment, to explain these observations, in addition to *in vitro* experiments.

## RESULTS

### Clinical data using peripheral blood samples

The baseline characteristics of the study participants are shown in Table 1. Among 46 controls and 28 patients with fragility HF (mean ages, 72.1 ± 5.3 [range, 65–90] and 78.3 ± 9.1 [range, 65–95] years, respectively), 31 (67.4%) and 21 (75.0%), respectively, were women. There were no significant differences in weight, height, body mass index (BMI), smoking status, alcohol intake, history of diabetes, and serum 25-hydroxyvitamin D_3_ (25-OH-D_3_) between the groups. Bone mineral density (BMD) values at the lumbar spine and proximal femurs were significantly lower in patients with HF than in those without HF. Serum C-terminal telopeptide of the type I collagen (CTX) levels were markedly higher in patients than in controls, whereas the difference in serum osteocalcin (OSC) levels between the groups was not statistically significant.

**Table 1:** Baseline characteristics of the study participants according to fragility HF status.

**Variables**	**Subjects without HF (*n* = 46)**	**Subjects with HF (*n* = 28)**	**P**
Sex, no. (%)			0.603
Female	31 (67.4)	21 (75.0)	
Male	15 (32.6)	7 (25.0)	
Age (years)	**72.1 ± 5.3**	**78.3 ± 9.1**	**0.002**
Weight (kg)	58.2 ± 8.9	56.7 ± 11.7	0.552
Height (cm)	154.4 ± 8.0	155.0 ± 8.6	0.751
BMI (kg/m2)	24.4 ± 3.2	23.6 ± 4.4	0.379
Current smoker, no. (%)	7 (15.2)	4 (14.3)	0.999
Alcohol intake ≥ 3 U/day, no. (%)	8 (17.4)	3 (10.7)	0.518
Diabetes, no. (%)	9 (19.6)	3 (10.7)	0.517
Serum 25-OH-D3 (ng/mL)^*^	22.7 ± 15.3	18.3 ± 9.7	0.177
BMD:			
Lumbar spine Z-score	**0.642 ± 0.160**	**−0.358 ± 1.486**	**0.005**
Femoral neck Z-score	**0.973 ± 1.455**	**−0.329 ± 0.934**	**< 0.001**
Total femur Z-score	**0.534 ± 0.926**	**−0.643 ± 0.975**	**< 0.001**
BTM:			
CTX (ng/mL) ^*^	**0.535 ± 0.229**	**0.786 ± 0.349**	**0.002**
Osteocalcin (ng/mL) ^*^	19.9 ± 8.6	21.3 ± 10.2	0.524

Values are presented as mean ± SD unless otherwise specified. **Bold** means that values are statistically significant. ^*^Serum CTX and OSC levels were measured using an electrochemical-luminescence immunoassay (Roche Diagnostics GmbH, Mannheim, Germany). Serum concentration of 25-OH-D3 was measured by radioimmunoassay (Cobra II Auto-γ Counting System; Packard Instruments, Downers Grove, IL).

The association of age with plasma ceramide levels was investigated by the Pearson correlation analysis (Supplementary Table 1). Age was positively correlated with circulating C16:0, C18:0, and C24:1 ceramide levels, while there was no association between age and plasma C14:0, C18:1, C20:0, and C24:0 ceramides.

**Figure 1:**
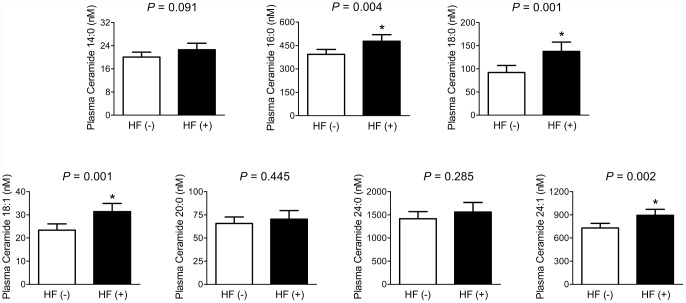
**Differences in plasma ceramide levels according to the fragility HF status.** After adjusting for confounders, the estimated means with 95% CIs were generated and compared using ANCOVA. ^*^Statistically significantly different from the control by ANCOVA. Multivariable confounding factors included sex, age, BMI, smoking status, alcohol intake, 25-OH-D_3_, and diabetes.

Difference in plasma ceramide levels according to fragility HF status was analyzed by analysis of covariance (ANCOVA) (Figure 1). After adjustment for potential confounders, including sex, age, BMI, smoking status, alcohol intake, 25-OH-D_3_, and diabetes, patients with fragility HF had 21.3%, 49.5%, 34.3%, and 22.5% higher plasma C16:0, C18:0, C18:1, and C24:1 ceramide levels than those without HF, respectively (statistically significant). On the other hand, there were no significant differences in plasma C14:0, C20:0, and C24:0 ceramide levels between the groups.

The risk for fragility HF according to plasma ceramide levels was determined through multiple logistic regression analysis (Table 2). After considering confounding factors, the odds ratios (ORs) per standard deviation (SD) increment in circulating C16:0, C18:0, C18:1, and C24:1 ceramide levels for fragility HF were 2.73, 3.20, 2.86, and 3.43, respectively. However, the ORs for HF in terms of plasma C14:0, C20:0, and C24:0 levels were not statistically significant.

**Table 2:** The risk for fragility HF according to plasma ceramide levels.

**Ceramide**	**OR (95% CIs) per SD increment in plasma ceramide levels**	***P***
C14:0	1.67 (0.91–3.05)	0.096
C16:0	**2.73 (1.34–5.57)**	**0.006**
C18:0	**3.20 (1.35–7.55)**	**0.008**
C18:1	**2.86 (1.45–5.63)**	**0.002**
C20:0	1.20 (0.68–2.11)	0.529
C24:0	1.34 (0.77–2.31)	0.298
C24:1	**3.43 (1.47–8.01)**	**0.004**

**Bold** means that values are statistically significant. Multivariable confounding factors included sex, age, BMI, smoking status, alcohol intake, 25-OH-D_3_, and diabetes. OR, odds ratio; CI, confidence interval; SD, standard deviation.

Multiple linear regression analyses were performed to examine the relationship of plasma ceramides with BMD and bone turnover markers (BTMs) (Table 3). After adjustment for sex, age, BMI, smoking status, alcohol intake, 25-OH-D_3_, and diabetes, plasma C18:0 and C24:1 ceramides were inversely associated with BMD values at the lumbar spine and total femur, respectively. However, other ceramides, including C14:0, C16:0, C18:1, C20:0, and C24:0, were not significantly correlated with BMD values at any site. Plasma C16:0, C18:0, C18:1, C24:0, and C24:1 ceramides, but not C14:0 and C20:0 levels, were positively related to serum CTX level, a bone resorption marker, whereas no circulating ceramides had a significant association with serum OCN level, a bone formation marker, in the multivariate adjustment model.

**Table 3:** The association of plasma ceramides with bone mineral density and bone turnover marker.

**Dependent variable**	**C14:0**		**C16:0**		**C18:0**		**C18:1**	
	β^*^	***P***	**β**	***P***	**β**	***P***	**β**	***P***
Lumbar spine *Z*-score	−0.166	0.232	−0.209	0.127	**−0.269**	**0.040**	−0.185	0.145
Femoral neck *Z*-score	−0.120	0.346	−0.039	0.762	−0.029	0.824	−0.146	0.236
Total femur *Z*-score	−0.186	0.134	−0.198	0.111	−0.222	0.076	−0.231	0.051
Serum CTX	0.221	0.068	**0.443**	**< 0.001**	**0.316**	**0.008**	**0.292**	**0.011**
Serum osteocalcin	−0.040	0.751	0.232	0.054	0.127	0.321	0.045	0.716
**Dependent variable**	**C20:0**		**C24:0**		**C24:1**			
	**β**	***P***	**β**	***P***	**β**	***P***		
Lumbar spine *Z*-score	0.027	0.840	−0.143	0.273	−0.244	0.114		
Femoral neck *Z*-score	0.184	0.134	0.214	0.080	−0.095	0.509		
Total femur *Z*-score	0.006	0.964	−0.013	0.913	**−0.355**	**0.010**		
Serum CTX	0.217	0.063	**0.236**	**0.043**	**0.497**	**< 0.001**		
Serum osteocalcin	0.043	0.729	0.121	0.328	0.222	0.060		

^*^Standardized coefficient. **Bold** means that values are statistically significant. Multivariable confounding factors included sex, age, BMI, smoking status, alcohol intake, 25-OH-D_3_, and diabetes.

### Clinical data using BM samples

To understand more accurate mechanisms explaining the detrimental effects of ceramides in human bone health, C16:0, C18:0, C18:1, and C24:1 ceramides, which showed the most convincing results in peripheral blood, were additionally measured in BM aspirates, directly reflecting the bone microenvironment.

Pearson correlation analysis showed that the peripheral blood and BM levels of all four ceramides, especially C18:0, C18:1, and C24:1, were significantly correlated (Supplementary Table 2).

**Figure 2:**
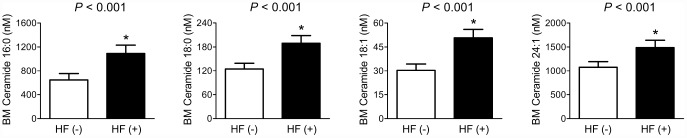
**Differences in BM ceramide levels according to the fragility HF status.** After adjusting for confounders, the estimated means with 95% CIs were generated and compared using ANCOVA. ^*^Statistically significantly different from the control by ANCOVA. Multivariable confounding factors included sex, age, BMI, smoking status, alcohol intake, 25-OH-D_3_, and diabetes.

After adjustment for sex, age, BMI, smoking status, alcohol intake, 25-OH-D_3_, and diabetes, patients with fragility HF had 69.2%, 51.9%, 67.3%, and 38.0% higher BM C16:0, C18:0, C18:1, and C24:1 ceramides levels than those without HF, respectively, consistent with the results using peripheral blood (Figure 2).

Finally, we investigated the association of ceramide levels with bone biochemical markers measured in BM samples. In the multivariate adjustment model, higher BM C16:0, C18:0, C18:1, and C24:1 ceramides levels were significantly associated with higher BM tartrate-resistant acid phosphatase-5b (TRAP-5b) levels, whereas only C18:0 ceramide was inversely correlated with the BM bone-specific alkaline phosphatase (BSALP) levels (Table 4). The BM C16:0, C18:0, and C24:1 ceramides levels had a positive association with the BM receptor activator of nuclear factor-κB ligand (RANKL) levels, and the BM C18:0 ceramide levels showed an inverse correlation with the BM osteoprotegerin (OPG) levels. Consequently, higher levels of all four ceramides measured in BM aspirates were significantly associated with higher BM RANKL/OPG ratio after adjustment for confounders.

**Table 4:** The association of various bone-related markers with the ceramide levels measured in BM plasma.

**Dependent variable**	**BM C16:0**		**BM C18:0**		**BM C18:1**		**BM C24:1**	
	β^*^	***P***	**β**	***P***	**β**	***P***	**β**	***P***
BM aspirates								
TRAP-5b	**0.476**	**< 0.001**	**0.306**	**0.018**	**0.405**	**0.001**	**0.446**	**< 0.001**
Bone-specific ALP	0.019	0.881	**−0.272**	**0.037**	−0.111	0.389	0.065	0.629
RANKL	**0.268**	**0.038**	0.240	0.068	**0.363**	**0.004**	**0.326**	**0.013**
OPG	−0.039	0.738	**−0.295**	**0.011**	−0.185	0.107	−0.151	0.207
RANKL/OPG ratio	**0.274**	**0.033**	**0.304**	**0.020**	**0.402**	**0.001**	**0.357**	**0.006**

^*^Standardized regression coefficient. The levels of TRAP-5b, bone-specific ALP, RANKL, OPG, and RANKL/OPG ratio were log-transformed because of their skewed distributions. **Bold** means that values are statistically significant. Multivariable confounding factors included sex, age, BMI, smoking status, alcohol intake, 25-OH-D_3_, and diabetes.

### *vitro* data

To further support our observations in humans, we treated the lineages of osteoclasts and osteoblasts with the ceramides within the *in vitro* system. Among various ceramides, we specifically focused on C18:0 and C24:1 ceramides, because these were related most strongly to fragility HF (Table 2) as well as age (Supplementary Table 1) in humans.

**Figure 3:**
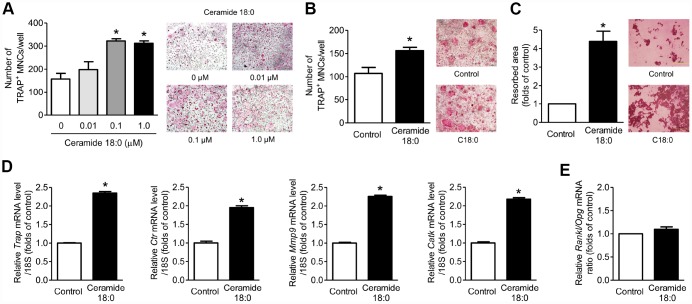
**Ceramide 18:0 stimulates osteoclast differentiation.** (**A**) Primary mouse BMMs were incubated with 30 ng/mL M-CSF and 100 ng/mL RANKL in the absence or presence of the indicated concentration of C18:0 for four days. After staining cells with TRAP, the number of TRAP-positive multinucleated cells (MNCs) (≥3 nuclei/cell) was determined to assess osteoclast differentiation. (**B**) Mouse BMMs were cocultured with primary calvaria osteoblasts for 10 days in medium containing 10^−8^ M 1α,25-OH(2) D_3_ and 10^−6^ M prostaglandin E2 without or with 0.1 μM C18:0. (**C**) Mouse BMMs were cultured with 30 ng/mL M-CSF and 100 ng/mL RANKL on dentine discs in the absence or presence of 0.1 μM C18:0 for 10 days. Resorption pits were visualized by staining with hematoxylin. (**D**) qRT-PCR expression analysis of osteoclast differentiation markers in mouse BMMs exposed to 30 ng/mL M-CSF and 100 ng/mL RANKL in the absence or presence of 0.1 μM C18:0 for 4 days. (**E**) qRT-PCR analysis to determine relative *Rankl* and *Opg* expression in mouse calvaria osteoblasts exposed to 50 μg/mL ascorbic acid and 10 mM β-glycerophosphate in the absence or presence of 0.1 μM C18:0 for 7 days. *Scale bars*: 500 μm for (**A**–**C**). Data are presented as mean ± SEM. ^*^P < 0.05 vs. untreated control using the Mann-Whitney *U* test or ANOVA followed by Tukey’s posthoc analysis.

When primary mouse BM macrophages (BMMs) were treated with or without various concentrations of C18:0 ceramide in the presence of macrophage colony-stimulating factor (M-CSF) and RANKL, C18:0 markedly stimulated the formation of differentiated osteoclasts, as shown by the TRAP staining results (Figure 3A), and increased osteoclastogenesis by C18:0 also was observed in the coculture system with mouse calvaria osteoblasts (Figure 3B). In addition, C18:0 enhanced bone resorption by approximately 4.4-fold compared to the untreated controls (Figure 3C). Consistently, the expression levels of osteoclast differentiation markers, such as *Trap*, *Ctr*, *Mmp9*, and *Catk*, were significantly increased by C18:0 (Figure 3D). To determine if the effects of C18:0 on osteoclasts were mediated by the production of RANKL and/or OPG in osteoblasts, primary mouse calvaria osteoblasts were incubated with or without C18:0 ceramide; however, there were no differences in the RANKL/OPG ratio between the two groups (Figure 3E), indicating that C18:0 ceramide ay directly stimulate osteoclastogenesis and the resultant bone resorption rather than through the increase of RANKL/OPG ratio from osteoblasts, at least within the *in vitro* system. Next, when we assessed the effects of C18:0 on osteoblast biology, the viability (Supplementary Figure 1A), ALP activity (Supplementary Figure 1B), and expression of osteoblast differentiation markers (Supplementary Figure 1C) were not changed by C18:0 treatment in mouse calvaria osteoblasts.

**Figure 4:**
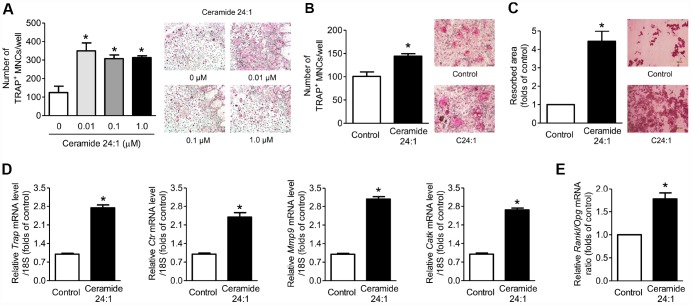
**Ceramide 24:1 stimulates osteoclast differentiation.** (**A**) Primary mouse BMMs were incubated with 30 ng/mL M-CSF and 100 ng/mL RANKL in the absence or presence of the indicated concentration of C24:1 for 4 days. After staining cells with TRAP, the number of TRAP-positive MNCs (≥ 3 nuclei/cell) was determined to assess osteoclast differentiation. (**B**) Mouse BMMs were cocultured with primary calvaria osteoblasts for ten days in a medium containing 10^−8^ M 1α,25-OH(2) D_3_ and 10^−6^ M prostaglandin E2 without or with 0.01 μM C24:1. (**C**) Mouse BMMs were cultured with 30 ng/mL M-CSF and 100 ng/mL RANKL on dentine discs in the absence or presence of 0.01 μM C24:1 for ten days. Resorption pits were visualized by staining with hematoxylin. (**D**) qRT-PCR expression analysis of osteoclast differentiation markers in mouse BMMs exposed to 30 ng/mL M-CSF and 100 ng/mL RANKL in the absence or presence of 0.01 μM C24:1 for 4 days. (**E**) qRT-PCR analysis to determine relative *Rankl* and *Opg* expression in mouse calvaria osteoblasts exposed to 50 μg/mL ascorbic acid and 10 mM β-glycerophosphate in the absence or presence of 0.01 μM C24:1 for seven days. *Scale bars*: 500 μm for (**A**–**C**). Data are presented as mean ± SEM. ^*^P < 0.05 vs. untreated control using the Mann-Whitney *U* test or ANOVA followed by Tukey’s posthoc analysis.

As observed in C18:0 treatment in the *in vitro* system, C24:1 ceramide also increased the osteoclastogenesis (Figures 4A, 4B), bone resorption (Figure 4C), and expression levels of osteoclast differentiation markers Figure 4D), and its effects on viability (Supplementary Figure 2A), ALP activity (Supplementary Figure 2B), and expression of osteoblast differentiation markers (Supplementary Figure 2C) were not observed in mouse calvaria osteoblasts. However, C24:1 treatment significantly increased the RANKL/OPG ratio in osteoblasts (Figure 4E), suggesting that C24:1 ceramide can stimulate bone resorption not only by the direct increase in osteoclast differentiation, but also the indirect regulation of RANKL/OPG system in osteoblasts in the *in vitro* system.

The *in vitro* experiments above were performed using BMMs isolated from young (6-weeks old) mice, as is widely used in bone research [15]. In addition to these, we investigated the effects of C18:0 and C24:1 ceramides on osteoclast differentiation from BMMs collected from old (24-months old) mice and confirmed that both ceramides markedly increased osteoclastogenesis (Supplementary Figure 3).

## DISCUSSION

It is well established that circulating ceramides increase with age [3, 5, 8] and aging is a primary risk factor for HF. In addition, ceramides have been shown previously to inhibit osteoblasts [9, 10] and to induce cell death in BMSCs [3]. Based on these backgrounds, we investigated the relationship between circulating levels of various ceramides with age and HF in humans. Our results confirm previous findings [5, 8, 16] that circulating ceramides show a positive correlation with age. We also found that certain ceramide species, such as C18:0 and C24:1, are significantly elevated in HF patients compared to controls after considering age. Moreover, the OR for fragility HF is highest in those patients with the highest levels of C24:1 ceramide, further underscoring the relationship between blood ceramide and HF. These data suggested that specific ceramides, such as C24:1 ceramide, could potentiallyserve as circulating biomarkers related to fracture risk. At the very least, our data showing elevated plasma ceramides in older subjects and in HF patients confirmed the results of previous studies linking increased ceramide with age-associated morbidity and mortality [4–7]. The pathogenesis of age-associated degenerative diseases reveals that changes in one organ system, such as bone or muscle, may occur alongside degenerative changes in other tissues, such as the brain. For example, loss of muscle strength and bone mass with aging has been observed in patients with cognitive impairment and Alzheimer’s disease [17–20]. Our data and those of others [5–7] suggested that age-related changes in systemic, circulating factors, such as C18:0 and C24:1 ceramide, may have important roles in the degenerative changes that occur in multiple tissues and organ systems with aging.

Our data point to a key role for ceramides in driving bone resorption in human bone metabolism. Elevated plasma ceramide levels in HF patients were independently associated with increased serum CTX, a marker of bone resorption, but no significant changes in serum OSC, a marker of bone formation. We further explored a potential role for ceramide in bone resorption using in *in vitro* approaches. These experiments demonstrated that C18:0 ceramide increased osteoclast differentiation and activity but did not increase RANKL secretion by osteoblasts. In contrast, C24:1 ceramide also increased osteoclast differentiation and activity but stimulated RANKL production by osteoblasts. Thus, ceramides may increase bone resorption by directly acting on osteoclast precursors or by acting indirectly through the production of RANKL by osteoblasts. The in *in vitro* experiments are consistent with our observations from the patient sample showing that markers of bone formation were not significantly altered with increased ceramide levels. Specifically, we saw no changes in osteoblast viability, alkaline phosphatase activity, or the expression of osteogenic genes with C18:0 or C24:1 ceramide treatment *in vitro*. These findings are novel, in that previous work has suggested the primary effects of ceramide on bone were related to their role in inducing cell death in either osteoblasts or BM stem cells [3, 9–13], and demonstrated that ceramides may negatively impact bone metabolism by stimulating osteoclasts as well as perhaps inhibiting osteoblasts.

The major strength of our study is that we measured the ceramides and bone biochemical markers in BM aspirates as well as in peripheral blood. Previous studies reported the weak association between blood and BM plasma levels of TRAP-5b, BSALP, and OPG, and even no detection of RANKL in peripheral blood [21]. Therefore, there have been great concerns that the measurement of these markers in peripheral blood may not adequately elucidate the underlying mechanisms of potential effectors on human bone metabolism [21, 22]. To alleviate this concern, we directly collected BM samples and demonstrated that higher ceramide levels were significantly associated with higher TRAP-5b levels in this human mechanism cohort. These are consistent with the results from *in vitro* experiments, and thus strongly support the role of ceramides, especially C18:0 and C24:1, as potential stimulators of osteoclastogenesis that may contribute to poor bone health.

Circulating ceramide is produced and secreted primarily by the liver, and it can be generated in the liver either by de novo synthesis or hydrolysis of sphingomyelin [23–25]. We showed previously that elevated C24:1 ceramide in circulating exosomes with age is due at least partly to increased nSMase2 activity in the liver [3]. nSMase, in turn, is thought to be increased in cells after exposure to oxidative stress [26] or to the inflammatory cytokine TNF-α [27], both of which are known to increase with age. Earlier reports indicated that ceramides, such as C2Cer, could suppress bone resorption by mature osteoclasts by inhibiting F-acting ring formation [28], suggesting that ceramide may act to suppress bone loss. Ceramides are phosphorylated by ceramide kinase to produce ceramide-1-phosphate (C1P) [29]. Though C1P has itself been observed to inhibit bone resorption by mature osteoclasts [28], an extensive body of literature exists supporting a role for C1P in macrophage survival, proliferation, and chemotaxis [29]. C1P is considered a potent pro-inflammatory agent, and many of its actions can be antagonistic to ceramides themselves. Thus, phosphorylation of ceramide by ceramide kinase is one potential pathway through which ceramides stimulate bone resorption by enhancing the survival, migration, and proliferation of osteoclast precursors. Future research may be directed at better understanding the balance between ceramide and C1P activity in the marrow microenvironment with aging, and how this relationship might be targeted to improve bone health and prevent bone loss.

Several potential limitations should be considered when interpreting our data. Importantly, because this was a case-control study, we could not determine whether a causal relationship existed between ceramide levels and osteoporosis-related phenotypes. As one effort to overcome this limitation, we additionally performed *in vitro* experiments with direct treatment of ceramides to bone cells. Second, because of the difficulties in obtaining BM samples, our sample size was relatively small. However, as described above, this is a unique cohort from which blood and BM samples were collected simultaneously. Finally, our study population consisted of patients who visited a referral hospital, and therefore may not be representative of the general population. This limitation may have resulted in selection bias.

In conclusion, we demonstrated that higher blood C16:0, C18:0, C18:1, and/or C24:1 levels were associated with increased risk of fragility HF, lower bone mass, and higher serum CTX levels in subjects 65 years or older. Furthermore, higher BM C16:0, C18:0, C18:1, and C24:1 ceramide levels were significantly correlated with higher BM TRAP-5b levels, and the effects of C18:0 and C24:1 ceramides on osteoclast differentiation were validated in *in vitro* systems. These results suggested that the association of increased ceramides, especially C18:0 and C24:1, with adverse bone phenotypes in the elderly could be explained mainly by the increase in osteoclastogenesis and bone resorption in humans.

## MATERIALS AND METHODS

### Study participants

The study population comprised consecutive patients 65 years or older who underwent hip surgery at the Department of Orthopedic Surgery, Asan Medical Center (AMC; Seoul, Korea) between November 2012 and December 2013. All patients underwent hip surgery because of fragility HF or other causes, such as osteoarthritis. Fragility fractures result from mechanical forces that would not ordinarily cause fracture, known as low-level (or low-energy) trauma [30, 31]. The World Health Organization has quantified this as forces equivalent to falls from a standing height or less [32]. Exclusion criteria were drugs that could affect bone metabolism taken for >6 months or within the previous 12 months before hip surgery, such as bisphosphonate, systemic glucocorticoids, or hormone replacement therapy; diseases that might cause secondary osteoporosis, such as hyperthyroidism or rheumatoid arthritis; fever (oral temperature ≥ 38.0°C) or abnormal complete blood count findings of leukocytes (<4.0 or >10.0 × 10^9^/L) or platelets (<150 or >350 × 10^9^/L); and abnormal liver or kidney functions. These criteria were used to eliminate systemic illness. Finally, subjects with fractures not caused by low-energy trauma, such as motor vehicle accidents or falls above a standing height, were also excluded. Among the 102 eligible participants not satisfying any of the detailed exclusion criteria, we could contemporaneously collect blood and BM samples during hip surgery from 74 participants with their consent. Consequently, 46 controls and 28 patients without and with fragility HF, respectively, were finally enrolled in our study.

A patient questionnaire was used to assess smoking (current smoker), alcohol intake (≥3 U/day), history of medication use, previous medical or surgical procedures, and reproductive status (including menstruation). This study was approved by the AMC institutional review board. Written informed consent was provided by all enrolled participants.

### Measurement of ceramides in peripheral blood and BM aspirates in humans

Venous blood and BM samples were collected during hip surgery. After sample centrifugation at 3000 revolutions per minute (rpm) for 5 minutes at 4°C, we carefully collected the supernatants to exclude cell components. All samples with hemolysis or clotting were discarded. Human peripheral and BM plasma were mixed well, and internal standard solution (500 nM C17 ceramide) was added to the samples before extraction. Ceramides were extracted by Bligh and Dyer method [33]. After lipid extraction, organic solutions containing lipids were dried using a vacuum centrifuge and stored at −20°C until liquid chromatography-tandem mass spectrometry (LC-MS/MS) analysis. The dried matter was reconstituted with methanol (MeOH) and injected into the LC-MS/MS system. All lipid standards, including internal standards, were purchased from Avanti-Polar Lipids (Alabaster, AL, USA) and Sigma-Aldrich Corp. (St. Louis, MO, USA).

Lipid levels were determined using an LC-MS/MS system equipped with 1290 HPLC (Agilent, Waldbronn, Germany) and QTRAP 5500 (AB Sciex, Toronto, Canada). A reverse-phase column (Pursuit5 C18, 150 × 2.1 mm) was used with mobile phase A (5 mM ammonium formate/MeOH/tetrahydrofuran [500/200/ 300]) and mobile phase B (5 mM ammonium formate/MeOH/ tetrahydrofuran [100/200/700]). The LC was run at 200 μL/minute and 35°C. The LC gradient was as follows: 50% of A at 0 minutes, 50% of A for 5 minutes, 50%–30% of A for 3 minutes, 30% of A for 7 minutes, 30%–10% of A for 7 minutes, 10% of A for 3 minutes, 10%–50% of A for 0.1 minutes, and 50% of A for 4.9 minutes. Multiple reaction monitoring was performed in the positive ion mode, and the extracted ion chromatogram corresponding to the specific transition for each lipid was used for quantification. The calibration range for each lipid was 0.1–1000 nM (*r*^2^ ≥ 0.99). Data were analyzed using Analyst 1.5.2 software.

### Biochemical measurement in humans

To measure biochemical BTMs, fasting blood samples were obtained in the morning. Serum CTX levels were measured using an electrochemical-luminescence immunoassay (Roche Diagnostics GmbH, Mannheim, Germany), with intra- and inter-assay coefficients of variation (CVs) of 1.0%–4.6% and 1.6%–4.7%, respectively. Serum OSC concentration was also measured using an electrochemical-luminescence immunoassay (Roche Diagnostics GmbH), with intra- and inter-assay CVs of 1.2%–4.0% and 1.7%–6.5%, respectively. Serum concentration of 25-OH-D_3_ was measured by radioimmunoassay (Cobra II Auto-γ Counting System; Packard Instruments, Downers Grove, IL), with a lower limit of detection of 0.6 ng/mL (1.5 nmol/L) and intra- and inter-assay CVs of < 3.5%.

The following bone biochemical markers were measured in BM samples with immunoassay kits: TRAP-5b (Cat #SB-TR201A; Immunodiagnostic Systems, Scottsdale, AZ, USA), BSALP (Cat #MBS262250; Mybiosource, San Diego, CA, USA), OPG (Cat #ab100617; Abcam, Cambridge, MA, USA), and RANKL (Cat #K1016; Immunodiagnostic Systems). The intra- and inter-assay CVs for each assay were as follows: TRAP-5b <9% and <9%, BSALP <7% and <12%, OPG <10% and <12%, and RANKL <3.5% and <9.3%, respectively.

### BMD measurement in humans

Areal BMD (g/cm^2^) was measured at the lumbar spine (L1–L4) and proximal femurs (femoral neck and total femur) by dual-energy x-ray absorptiometry using the Lunar system (Prodigy; Madison, WI, USA) software version 9.30.044. The precision of the equipment, presented as CVs, was 0.67% and 1.25% for the lumbar spine and femoral neck, respectively, in 17 volunteers who were not enrolled in the study. Each volunteer underwent five scans on the same day, getting on and off the table between examinations. Bone density was expressed as the Z-score, which is defined as the number of SDs above or below mean for healthy subjects of the same sex and age.

### Reagents for *in vitro* experiments

C18:0 and C24:1 ceramides were purchased from Cayman Chemical (Ann Arbor, MI, USA). Soluble RANKL was purchased from R&D System, Inc. (Minneapolis, MN, USA), and M-CSF was purchased from PeproTech EC (London, UK). Fetal bovine serum (FBS) and α-minimum essential medium (α-MEM) were purchased from Gibco (Grand Island, NY, USA) and Wel Gene (Daegu, Korea), respectively. Prostaglandin E_2_ and 1α,25-dihydroxyvitamin D_3_ (1α,25-OH[2] D_3_) were purchased from Sigma-Aldrich Corp.

### Cell cultures

Primary osteoclasts were generated as previously described [15]. Primary mouse BM cells (BMCs) were obtained by flushing the femur and tibia of 6-week-old ICR or 24-month-old C57/BL6 mice (Orient, Seongnam, Korea) and cultured at 37°C in α-MEM containing 10% FBS, 100 U/mL penicillin, and 100 μg/mL streptomycin in a humidified atmosphere with 5% CO_2_. After a 24-hour culture, nonadherent cells were collected and cultured in 96-well plates at a density of 4 × 10^4^ cells/well. The BMMs were induced to differentiate into osteoclasts with 30 ng/mL M-CSF and 100 ng/mL soluble RANKL for more than four days, with the culture medium being changed every two to three days.

Primary mouse osteoblasts were isolated by sequential collagenase digestion of calvaria obtained from newborn mice and maintained in α-MEM containing 10% FBS, 100 U/mL penicillin, and 100 μg/mL streptomycin. Mature osteoblasts were generated from mouse calvaria osteoblasts in the presence of 50 μg/mL ascorbic acid and 10 mM β-glycerophosphate for seven days.

For the coculture system, primary mouse calvaria osteoblasts were cultured for one day in 48-well plates at a density of 1 × 10^4^ cells/well. Primary mouse BMMs (1 × 10^5^ cells) were added to each well, and BMMs were cocultured with osteoblast for ten days in the presence of 10^−8^ M 1α,25-OH(2) D_3_ and 10^−6^ M prostaglandin E2, changing the culture medium every two to three days.

### TRAP staining and *in vitro* resorption assay

After culture in an osteoclast-inducing medium, adherent cells were fixed and stained with TRAP, an enzymatic marker of osteoclasts, using a leukocyte acid phosphatase kit (Sigma-Aldrich Corp.) in accordance with the manufacturer’s instructions. TRAP-positive multinucleated cells containing ≥3 nuclei were considered to be osteoclasts and were counted under a light microscope (Olympus Optical; Tokyo, Japan).

To measure the resorption area, BMMs (4 × 10^4^ cells/well in 96-well plates) were seeded onto dentine discs with 30 ng/mL M-CSF and 100 ng/mL soluble RANKL. After 10 days, the cells on the dentine discs were removed completely by wiping with a cotton swab, and then the dentine slices were stained with hematoxylin (Sigma-Aldrich Corp.) for one minute. The area of resorbed pits was analyzed using Image-Pro Plus software (MediaCybernetics, Silver Spring, MD, USA).

### Quantitative reverse-transcription PCR (qRT-PCR)

Total RNA was isolated using TRIzol reagent (Invitrogen, Carlsbad, CA, USA), according to the manufacturer's protocol. First-strand cDNA was synthesized with the Superscript III First-Strand Synthesis System (Invitrogen) using oligo dT primers. qRT-PCR was performed using the Light Cycler 480 (Roche; Mannheim, Germany) with Probes Master Mix (Roche). The primers were *Trap* (Mm00475698_m1), *Ctr* (Mm00432282_m1), *Mmp9* (Mm00442991_m1), *CatK* (Mm00484039_m1), *Rankl* (Mm00441906_m1), *Opg* (Mm01205928_m1), *Col1* (Mm00801666_g1), *Alp* (Mm00475834_m1), and *Ocn* (Mm03413826_mH) from Applied Biosystems (Foster city, CA, USA). The threshold cycle (Ct) value for each gene was normalized to the Ct value of 18S (Hs03928990_g1).

### Cell viability

Cell viability was measured using the Cell Counting Kit-8 (Dojindo; Kumamoto, Japan), according to the manufacturer’s instructions. Briefly, 10 μL WST-8 dye (2-[2-methoxy-4-nitrophenyl]-3-[4-nitrophenyl]-5-[2,4-disulfophenyl]-2H-tetrazolium, monosodium salt) was added to each well in a 96-well plate for one hour at 37°C, and the absorbance at 450 nm was then read using a microplate reader SPECTRAmax 340PC (Molecular Devices, Palo Alto, CA, USA) with a reference wavelength of 650 nm.

### ALP activity

Primary mouse osteoblasts were seeded at a density of 5 × 105 cells/well in 12-well plates and were differentiated into osteoblasts for seven days. The cells were washed with PBS, and the ALP activity was measured using the p-nitrophenyl phosphate hydrolysis method [34]. The ALP activity in each sample was normalized relative to total cellular protein content, which was determined by the BCA method (Pierce, Rockland, IL, USA).

### Statistical analysis

All data are presented as means ± SD or as numbers and percentages, unless otherwise specified. The baseline characteristics of the study population according to the status of fragility HF were compared using Student’s *t*-tests for continuous variables and χ^2^ tests for categorical variables. Relationships between age and plasma ceramide levels and between peripheral plasma and BM ceramide levels were investigated using Pearson’s correlation analysis. The multivariable-adjusted least-square mean levels (95% confidence intervals [CIs]) of ceramides in peripheral blood and BM samples in terms of the presence of fragility HF were estimated and compared by ANCOVA after adjustment for potentially confounding factors, including sex, age, BMI, smoking status, alcohol intake, 25-OH-D_3_, and diabetes. These confounding variables were selected based on their clinical applicability. To generate ORs per SD increment in plasma ceramide levels for fragility HF, we performed multiple logistic regression analyses after adjustment for confounding variables. The associations of peripheral blood and BM ceramide levels with BMD, BTMs, and bone-related markers in BM were investigated through multiple linear regression analyses.

All *in vitro* data were expressed as means ± standard error of mean (SEM) of at least three independent experiments conducted with triplicate measurements, unless otherwise specified. The significance of differences between two groups was assessed using the Mann-Whitney *U* test, whereas differences between ≥ 3 groups were tested using the analysis of variance (ANOVA) with posthoc analysis via Tukey's honest significance test.

All statistical analyses were performed using SPSS version 18.0 (SPSS, Inc., Chicago, IL, USA). *P* < 0.05 was considered statistically significant.

## Supplementary Material

Supplementary Figures

Supplementary Tables
