# Supplementary material for: Elevated ceramides 18:0 and 24:1 with aging are associated with hip fracture risk through increased bone resorption
Source: Aging (Albany NY). 2019 Nov 1;11(21):9388–404. doi: 10.18632/aging.102389 (PMC6874435; doi:10.18632/aging.102389)
Supplement: Supplementary Figures [file aging-11-102389-s002.pdf]

SUPPLEMENTARY FIGURES

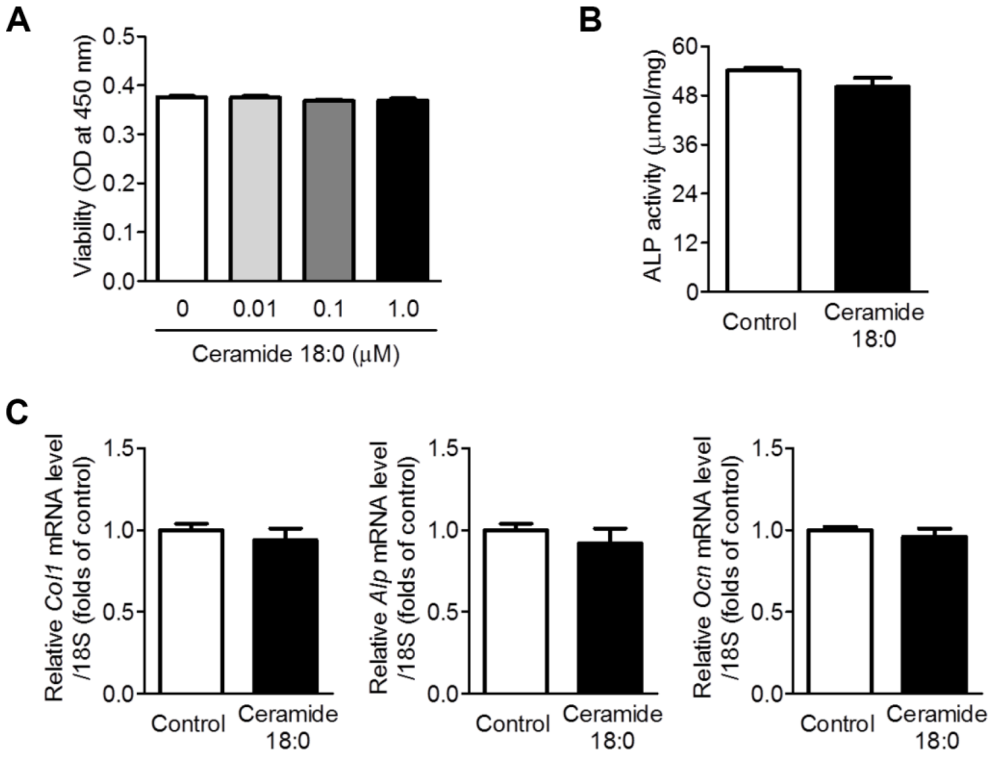

Supplementary Figure 1. No effects of ceramide 18:0 on osteoblast biology. (**A**) The viability of primary mouse calvaria osteoblasts was assessed using a cell counting kit-8 assay after exposure to the indicated concentration of C18:0 for 48 hours measured. (**B**) ALP activity of calvaria osteoblasts in medium containing 50 µg/mL ascorbic acid and 10 mM β-glycerophosphate without or with 0.1 µM C18:0 for seven days. The ALP activity was normalized by total cellular protein amounts. (**C**) Quantitative RT-PCR expression analysis of osteoblast differentiation markers in calvaria osteoblasts exposed to 50 µg/mL ascorbic acid and 10 mM β-glycerophosphate with or without 0.1 µM C18:0 for seven days. Data are presented as mean ± SEM.

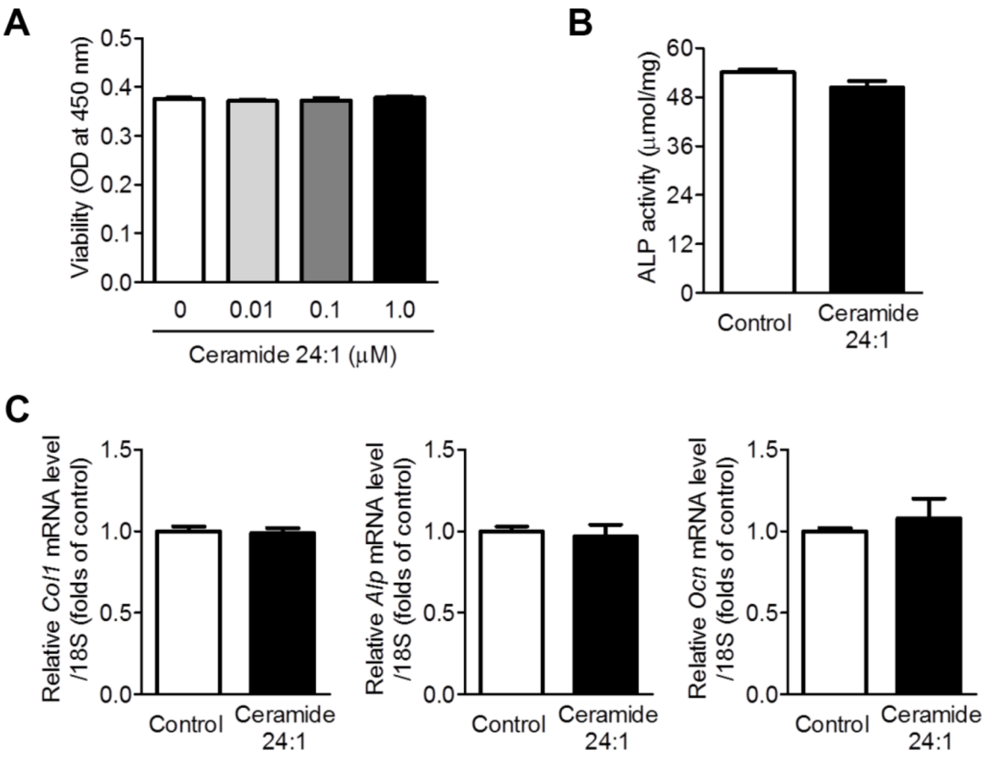

Supplementary Figure 2. No effects of ceramide 24:1 on osteoblast biology. (**A**) The viability of primary mouse calvaria osteoblasts was assessed using a Cell Counting Kit-8 assay after exposure to the indicated concentration of C24:1 for 48 hours measured. (**B**) ALP activity of calvaria osteoblasts in medium containing 50 μg/mL ascorbic acid and 10 mM β-glycerophosphate without or with 0.01 μM C24:1 for seven days. The ALP activity was normalized by total cellular protein amounts. (**C**) qRT-PCR expression analysis of osteoblast differentiation markers in calvaria osteoblasts exposed to 50 μg/mL ascorbic acid and 10 mM β-glycerophosphate without or with 0.01 μM C24:1 for seven days. Data are presented as mean ± SEM.

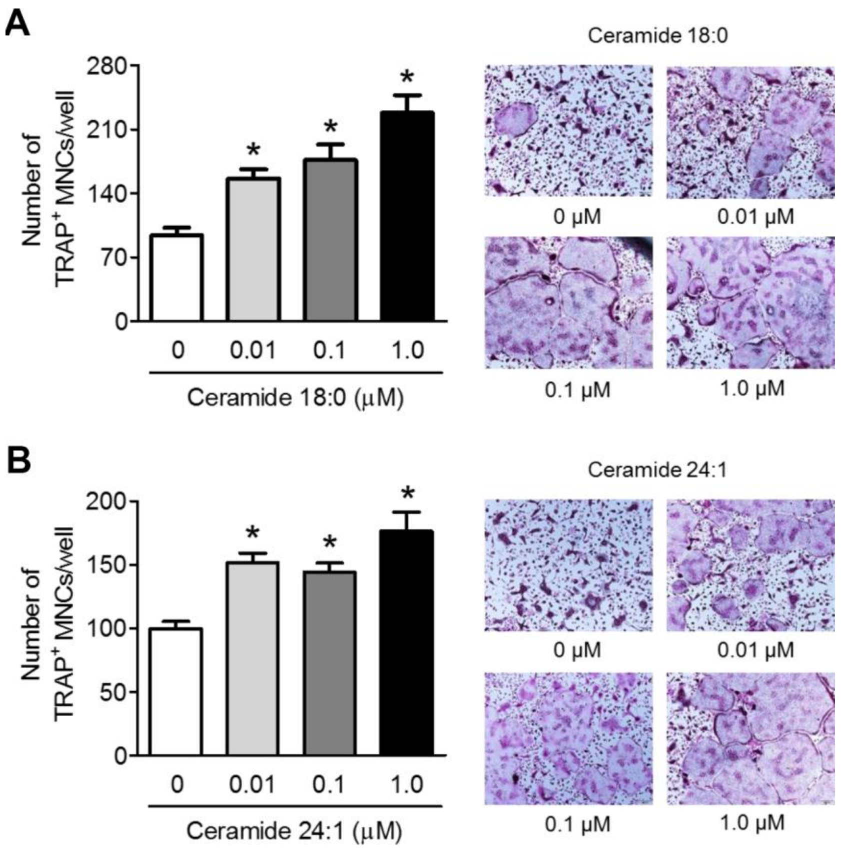

Supplementary Figure 3. Ceramide 18:0 and 24:1 stimulates osteoclast differentiation from BMMs isolated from old mice. Primary mouse BMMs were obtained from 24-month-old mice and incubated with 30 ng/mL M-CSF and 100 ng/mL RANKL in the absence or presence of the indicated concentration of C18:0 (A) and C24:1 (B) for four days. After staining cells with TRAP, the number of TRAP-positive multinucleated cells (MNCs) (≥3 nuclei/cell) was determined to assess osteoclast differentiation. *Scale bars*: 500 μm for (A) and (B). Data are presented as mean ± SEM. $^{*}P < 0.05$ vs. untreated control using the ANOVA followed by Tukey's posthoc analysis.
