# Supplementary material for: Elevated ceramides 18:0 and 24:1 with aging are associated with hip fracture risk through increased bone resorption
Source: Aging (Albany NY). 2019 Nov 1;11(21):9388–404. doi: 10.18632/aging.102389 (PMC6874435; doi:10.18632/aging.102389)
Supplement: Supplementary Tables [file aging-11-102389-s001.pdf]

SUPPLEMENTARY TABLES

Supplementary Table 1. The correlation between age and plasma ceramide levels.

Ceramide	γ*	*P*
C14:0	0.073	0.537
C16:0	**0.237**	**0.042**
C18:0	**0.243**	**0.037**
C18:1	0.053	0.656
C20:0	0.018	0.879
C24:0	−0.084	0.476
C24:1	**0.344**	**0.003**

*Pearson correlation coefficient. **Bold** means that values are statistically significant.

Supplementary Table 2. The correlation between peripheral blood and BM ceramide levels measured.

Ceramide	γ*	*P*
C16:0	**0.296**	**0.011**
C18:0	**0.660**	**<0.001**
C18:1	**0.505**	**<0.001**
C24:1	**0.522**	**<0.001**

*Pearson correlation coefficient. **Bold** means that values are statistically significant.
